# Single-cell sequencing reveals reversible glial remodeling in the visual cortex during visual deprivation and recovery

**DOI:** 10.3389/fimmu.2026.1730619

**Published:** 2026-02-18

**Authors:** Xiaoqi Gong, Jiaojiao Feng, Zhe Xu, Yunxiao Xie, Yibo Han, Jing Li, Guodong Tang, Yuxi Liu, Xiaoyun Dong, Shuhan Li, Jun Zhang, Junru Wang, Runxun Liu, Jike Song, Hongsheng Bi

**Affiliations:** 1 College of Ophthalmology and Optometry, Shandong University of Traditional Chinese Medicine, Jinan, China; 2 The First Clinical Medical College, Shandong University of Traditional Chinese Medicine, Jinan, China; 3 Affiliated Eye Hospital of Shandong University of Traditional Chinese Medicine, Jinan, China; 4 Shandong Academy of Eye Disease Prevention and Therapy, Shandong Provincial Key Laboratory of Integrated Traditional Chinese and Western Medicine for Prevention and Therapy of Ocular Diseases, Jinan, China

**Keywords:** form deprivation and recovery, microglia, oligodendrocytes, single-cell RNA sequencing, transcriptional reprogramming

## Abstract

**Introduction:**

The visual cortex exhibits remarkable experience-dependent plasticity, which can be profoundly disrupted by abnormal visual input. Form-deprivation myopia (FDM) is a well-established model for studying ocular growth; however, the specific responses and functional roles of non-neuronal cells in the visual cortex during both deprivation and recovery remain poorly understood. This study aimed to comprehensively characterize the dynamic alterations in these cells across the course of deprivation and subsequent visual restoration.

**Methods:**

We employed single-cell RNA sequencing (scRNA-seq) to delineate the transcriptomic landscape of the primary visual cortex (V1) in a guinea pig model. Two-week-old animals were assigned to three groups: normal control (NC), form-deprivation (FDM; 5 weeks of monocular deprivation), and recovery (REC; 4 weeks of deprivation followed by 1 week of restored vision). Key findings were validated using immunofluorescence, quantitative PCR, Western blotting, and transmission electron microscopy. Bioinformatic analyses, including trajectory inference and cell-cell communication mapping, were performed to elucidate cellular dynamics and interactions.

**Results:**

Visual deprivation induced a pronounced pro-inflammatory transformation in microglia compared with the NC group, characterized by significant upregulation of immune-related pathways such as IL-17, TNF-α, and Toll-like receptor signaling. Concurrently, oligodendrocyte numbers were markedly reduced in the FDM group, accompanied by myelin deficits and downregulation of the key transcription factor Zbtb16. Trajectory analysis revealed a blockade in oligodendrocyte differentiation, while intercellular communication analysis indicated enhanced inflammatory signaling from microglia to oligodendrocyte precursors. Notably, the recovery phase largely reversed these alterations: microglial inflammation was substantially attenuated, the expression of myelin-related genes such as Plp1 was restored, oligodendrocyte numbers and myelin integrity were restored to near-control levels, and the differentiation blockade was resolved.

**Conclusions:**

This study demonstrates that non-neuronal cells in the visual cortex, which include microglia and oligodendrocytes, undergo extensive yet reversible reprogramming in response to changes in visual input. These findings highlight a dynamic microglia–oligodendrocyte axis as a critical cellular mechanism underlying cortical plasticity in myopia, suggesting potential molecular targets for visual rehabilitation strategies.

## 1 Introduction

Form-deprivation myopia (FDM) is one of the most widely used experimental models for studying the mechanisms of myopia development (1). It induces axial elongation and myopic progression by degrading retinal image quality, thereby reducing contrast sensitivity and spatial frequency perception. These effects are reversible upon restoration of normal vision, highlighting that ocular growth is tightly regulated by visual feedback (2–4). Abnormal visual signals transmitted to the brain can accelerate myopic progression, triggering a cascade of neurobiological responses (5, 6).

The visual cortex, a major center for sensory processing, exhibits profound experience-dependent plasticity during early postnatal development (7). This plasticity allows neurons and circuits to dynamically remodel in response to environmental inputs. Disruption of normal visual experience during critical developmental periods—such as by congenital cataract (8), corneal opacity (9), or form deprivation (10)—can cause long-lasting alterations in cortical structure and function. Studies in cats and mice have demonstrated that monocular deprivation (MD) reduces neural activity and connectivity in cortical layers representing the deprived eye, while enhancing responses from the non-deprived eye (11–13). Remarkably, reopening the deprived eye restores cortical responsiveness, underscoring the brain’s capacity for recovery (14). Transcriptomic analyses further revealed that visual experience drives cell type–specific gene expression programs that are disrupted by deprivation but partially recover when normal vision returns, indicating that molecular reprogramming underlies experience-dependent cortical plasticity (15).

While neuronal mechanisms of cortical plasticity have been extensively characterized, growing evidence highlights essential roles for non-neuronal cells, particularly microglia (16) and oligodendrocytes (17). Microglia, the resident immune cells of the central nervous system, actively sculpt neural circuits by regulating synaptic pruning, maturation, and homeostasis (18). Disruption of microglial function, such as through P2Y12 receptor deficiency, abolishes ocular dominance plasticity following MD, demonstrating that microglial activity is indispensable for visual cortical remodeling (19). Researchers have discovered that the absence of microglia during critical periods alters the shift in ocular dominance induced by monocular deprivation, with significant impairment of cortical plasticity (20). Oligodendrocytes, through myelination, modulate the speed and synchrony of neuronal signaling and help consolidate mature circuits (21). Myelin-associated molecules, such as Nogo-A/B, restrict plasticity via NgR signaling, thereby limiting functional recovery under pathological conditions (22). Prolonged visual deprivation shortens myelin sheaths and alters oligodendrocyte morphology, changes potentially mediated by glutamate and axon-derived factors (23). Therefore, the expression levels of myelin structural proteins, such as *Plp1*, can serve as a sensitive indicator of oligodendrocyte functional status and cortical circuit maturity. Their dynamic changes help elucidate the mechanisms of experience-dependent myelin remodeling.

Recent studies have revealed a bidirectional regulatory axis between microglia and oligodendrocytes, whereby microglia influence oligodendrocyte lineage progression, myelination, and regeneration (24). This interaction plays a pivotal role in maintaining cortical homeostasis and responding to injury. However, the specific responses of these glial populations to visual deprivation and their contributions to cortical dysfunction and recovery in FDM remain poorly understood. The mechanisms by which microglial activation and inflammatory signaling affect cortical circuits (25), and how oligodendrocyte plasticity contributes to structural and functional recovery, represent important gaps in current knowledge (26).

To address these questions, we employed single-cell RNA sequencing (scRNA-seq) (27) to map the cellular and molecular landscape of the primary visual cortex in a guinea pig model of FDM. Our preliminary data revealed robust microglial activation following deprivation, characterized by upregulation of pro-inflammatory pathways, along with marked alterations in oligodendrocyte differentiation and myelin-related gene expression. Notably, these transcriptional changes were largely reversed after visual recovery, indicating that glial cells possess dynamic and reversible reprogramming capacity in response to sensory input.

In this study, we comprehensively characterize the molecular signatures of microglia and oligodendrocytes during deprivation and recovery using scRNA-seq, coupled with molecular, immunohistochemical, and ultrastructural validation. We aim to delineate the inflammatory and transcriptional pathways that drive glial plasticity, with a focus on key regulators such as Zbtb16, and to define how these mechanisms contribute to cortical homeostasis and experience-dependent remodeling. Our findings reveal a dynamic microglia–oligodendrocyte axis that orchestrates cortical responses to altered visual input, providing novel insights into the glial basis of cortical plasticity and identifying potential targets for visual rehabilitation strategies.

## 2 Materials and methods

### 2.1 Guinea pig model of monocular form deprivation and recovery

Two-week-old tricolour guinea pigs (Cavia porcellus), weighing 90–110 g, were sourced from a commercial supplier and housed in plastic cages (54.5 × 39.5 × 20 cm³) under controlled temperature and humidity conditions. Each cage contained five animals. The facility maintained a 12-hour light/dark cycle with an illuminance of approximately 350 lux. Food and water were provided twice daily, supplemented with fresh vegetables. All experimental protocols were approved by the Animal Care and Use Committee of the Shandong Eye Disease Prevention and Treatment Institute and conducted in compliance with the ARVO Statement for the Use of Animals in Ophthalmic and Vision Research.

**Figure 1:**
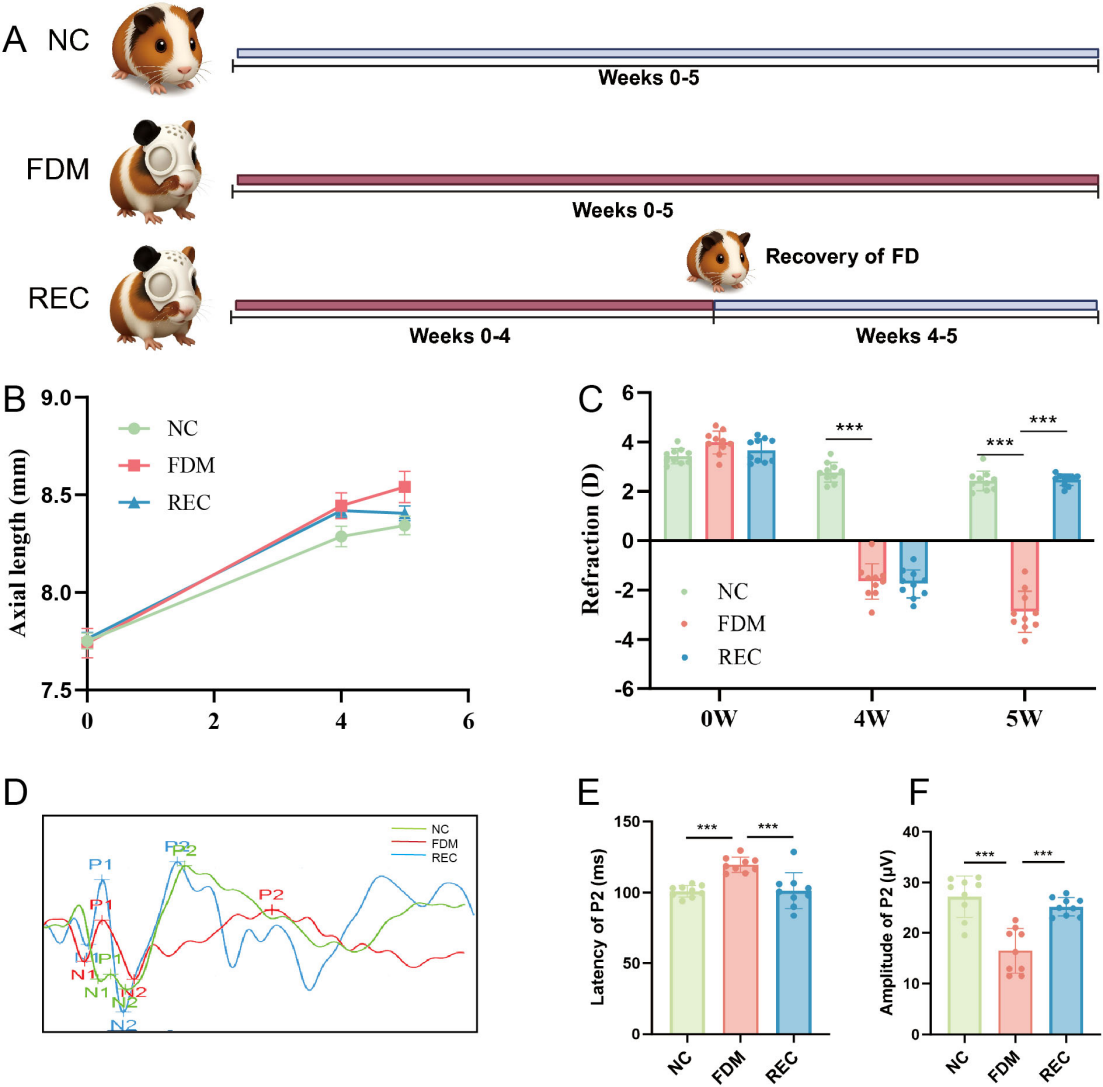
Changes in ocular biological parameters. **(A)** Experimental timeline for all three groups: Normal Control (NC, n=15), Form-Deprivation Myopia (FDM, n=15), and Recovery (REC, n=15). The FDM group underwent 5 weeks of monocular deprivation; the REC group underwent 4 weeks of deprivation followed by 1 week of visual restoration. **(B)** Growth curve of axial length from Week 0 to Week 5 (n = 15 per group). **(C)** Refractive error at Week 0 and Week 5 (n = 10 per group). ***P < 0.001, one-way ANOVA with Tukey’s HSD test. **(D)** Representative flash visual evoked potential (fVEP) traces at Week 5. Traces were selected to reflect the mean group differences in P2 latency and amplitude. **(E)** Quantification of P2 amplitude (n = 9 per group). **(F)** Quantification of P2 latency (n = 9 per group). Data in F and G are presented as mean ± SEM with individual data points superimposed; ***P < 0.001, one-way ANOVA with Tukey’s HSD test.

The guinea pigs were randomly assigned to three groups (n = 15 per group): an NC group, an FDM group, and a REC group (**Figure 1A**). The NC group received no intervention and was raised under standard visual conditions. In the FDM group, the right eye of each animal was covered with a 3D-printed shield made from modified latex balloon material, allowing 60% light transmittance (28). The shield was designed to fully occlude only the right eye while leaving the left eye, nose, mouth, and ears exposed. Animals in the REC group underwent four weeks of monocular form deprivation followed by one week of visual recovery under normal conditions. The shield did not come into contact with the cornea or eyelids. Shields were inspected three times per day during the 12-hour light phase to ensure proper placement and cleanliness, and were replaced as necessary to maintain fit and hygiene.

### 2.2 *In vivo* ocular phenotyping: refraction and axial length measurement

After myopia induction at 4 and 5 weeks, the headgear was removed and guinea pigs were dark-adapted for 12 hours prior to examination. To induce mydriasis, one drop of 0.5% compound tropicamide eye drops (Shenyang Xingqi Ophthalmic Co., Ltd., Shenyang, China) was administered into the conjunctival sac every 5 minutes for a total of three instillations. Refraction was measured 30–45 minutes after the last instillation using an infrared photorefractometer (Striatech GmbH, Germany), a high-precision instrument suitable for refractive assessment in small animals. At least six measurements were taken per eye, and the mean value was calculated as the final result.

Axial length was measured using an A-scan ultrasound system (Cinescan, Quantel Medical, France). Before measurement, topical anesthesia was induced by applying a single drop of 0.4% oxybuprocaine hydrochloride (Benoxicam, Santen Pharmaceutical, Japan) into the conjunctival sac. The probe frequency was set to 11 MHz, and ultrasound velocities were defined as follows: anterior chamber 1557 m/s, lens 1723 m/s, and vitreous 1540 m/s, based on established literature (29). Approximately two minutes after anesthesia, the probe was aligned perpendicularly to the corneal apex. Ten consecutive A-scan measurements were recorded per eye. Measurements with poor or non-standard waveforms were excluded, and the ten measurements with the clearest and most interpretable waveforms were retained for analysis. The mean of these ten values was used for statistical comparison.

### 2.3 Single-cell RNA sequencing and bioinformatic analysis

#### 2.3.1 Tissue dissociation and single-cell suspension preparation

After five weeks, all guinea pigs were euthanized via intraperitoneal injection of an overdose of pentobarbital sodium solution (100 mg/kg). Decapitation could be performed once corneal reflexes and pain responses had ceased. The skull was opened along the sagittal suture to expose the brain. A coronal section containing the V1 area was isolated using a brain matrix, and under a microscope, a 3 mm thick block of visual cortex was dissected from each hemisphere and rinsed in PBS.

Tissue samples were dissociated into single-cell suspensions using a two-step enzymatic digestion with freshly prepared Enzyme Mix 1 (Buffer Z + Enzyme P) and Enzyme Mix 2 (Buffer Y + Enzyme A). The digested tissue was filtered through a 70-μm strainer. No specific nuclear isolation step was performed. Cell suspensions were then subjected to debris removal using a Debris Removal Solution gradient centrifugation and erythrocyte lysis. Viable cells were isolated using Dead Cell Removal MicroBeads (Miltenyi Biotec) according to the manufacturer’s protocol. Cortical tissue samples from three animals within the same experimental group were pooled prior to library construction.

#### 2.3.2 Library preparation and sequencing using the 10x genomics platform

Single-cell RNA-seq libraries were constructed using the 10x Genomics Single Cell 3’ Gene Expression v3.1 platform, following the manufacturer’s instructions. Briefly, single cells were partitioned into Gel Bead-in-Emulsions (GEMs) for cell lysis, barcoding, and reverse transcription. The resulting cDNA was amplified, and libraries were prepared for sequencing. Libraries were quantified using an Agilent 4150 Bioanalyzer and a Qubit fluorometer, and sequenced on an Illumina NovaSeq 6000 system with a PE150 strategy.

#### 2.3.3 Data processing, integration, and clustering

Raw sequencing data were processed using the Cell Ranger pipeline (v3.1.0) aligned to the Cavpor3.0 reference genome. Downstream analysis was performed in R using the Seurat package (v4.3.0.1). Low-quality cells were filtered out based on UMI counts (<1000 or >8000), gene counts (<800), and high mitochondrial gene content (>5%). Data were normalized, and the top 2000 highly variable genes were selected. Batch effects were corrected using Harmony, and cells were clustered based on the first 15 corrected principal components. Results were visualized using UMAP.

#### 2.3.4 Cell type annotation and differential gene expression analysis

Cell clusters were annotated based on the expression of differentially expressed genes (DEGs), identified using a threshold of |log2FC| > 2 and an adjusted p-value < 0.001, compared to known cell-type markers. Gene Ontology (GO) enrichment analysis for biological processes was performed on cluster-specific DEGs using the clusterProfiler package.

### 2.4 Cortical function assessment by flash visual evoked potentials

Flash visual evoked potentials (FVEP) were recorded at Week 5 using an OPTOPROBE ophthalmic imaging system (Optoprobe OPTO-III, Optoprobe Sciences Ltd., UK). Prior to testing, guinea pigs were dark-adapted for 12 hours, and all electrophysiological procedures were performed under red light illumination. Animals were placed in a stabilized position on the surgical table with subcutaneous electrodes positioned as follows: the active electrode over the occipital cortex, the reference electrode on the cheek, and the ground electrode on the tail.

A flash stimulus at a frequency of 1 Hz was delivered, with each recording session consisting of 60 flashes. Upon completion of the test, the latency and amplitude of the P2 wave (a positive waveform component occurring approximately 100–150 ms post-stimulus, reflecting cortical processing) were recorded for subsequent analysis.

### 2.5 Expression validation by quantitative PCR

Following a five-week intervention period, three guinea pigs were randomly selected from each group for PCR analysis. Total RNA was isolated from visual cortex tissue using Trizol (Thermo Fisher Scientific, USA). cDNA was synthesized for quantitative polymerase chain reaction using HiScript II QRT SuperMix. qPCR was performed on the StepOnePlus real-time fluorescent quantitative PCR system using Hieff qPCR SYBR Green Premix. Primer sequences were as follows: Guinea pig ITPA forward: GAACACAGACCGGTAGAGGG; reverse: GTATCCTCCACCAGAACGGG; Guinea pig ZFP36 forward: GGGAATCTGGAGCCTGAACTC, reverse: TGGAGGTCGTAGTTGGTGAGG.

### 2.6 Immunofluorescence staining and microglial morphological analysis

Following perfusion fixation, brain tissues were post-fixed in 4% paraformaldehyde (PFA). One set of brains was fixed for 12–16 hours before transfer to 30% sucrose, while another set was fixed for 2 hours, rinsed in cold PBS, and subsequently cryoprotected in 30% sucrose. Coronal sections were cut at a thickness of 10–20 μm using a cryostat.

The sections were permeabilized with 0.25% Triton X-100 and blocked with 5% bovine serum albumin (BSA) and 3% normal goat serum. They were then incubated with a primary antibody against Iba1 (rabbit, 1:500) at 4 °C for 24–48 hours. After washing with PBS, the sections were incubated with an Alexa Fluor 488-conjugated secondary antibody (1:500) for 2 hours at room temperature. Nuclei were stained with DAPI. Finally, the sections were washed, mounted with anti-fade medium (Fluoro-Gel, EMS), and imaged using a Leica SP8 confocal microscope.

The visual field was randomly selected from the primary visual cortex (V1 area). Three non-overlapping fields of view were captured per animal. All imaging parameters, including laser intensity, gain, and exposure time, were maintained consistently across all specimens and experimental sessions. For the quantification of glial cell density, to exclude effects of cell density or slice thickness variations, Iba1+ signal intensity was normalized against the number of DAPI-positive nuclei within each field of view.

To visualize morphological changes, two-dimensional skeleton reconstructions of Iba1+ microglia were generated using the “Skeletonize” and “Analyze Skeleton” plugins in ImageJ (Fiji).

### 2.7 Protein quantification by digital capillary western blotting

The protein expression levels of *ITPA, ZFP36, Zbtb16*, and β-actin were analyzed using a ProteinSimple Abby capillary western blot system (ProteinSimple, CA, USA). Three visual cortex samples from each group were randomly selected for protein extraction. Tissues were homogenized on ice in RIPA lysis buffer containing PMSF protease inhibitor, using a tissue-to-buffer ratio of 10 mg:100 µL. The homogenates were centrifuged at 8000 rpm for 5 minutes at 4°C, and the resulting supernatants were collected. Protein concentration was determined using a BCA protein assay kit (Beyotime Bio, Shanghai, China). Each sample was diluted with PBS (1X) to a final concentration of 2 mg/mL.

Capillary western blot was performed on the Abby system according to the manufacturer’s instructions. The following primary antibodies were used: The following primary antibodies were used: ITPA (1:50, 16134-1-AP, Proteintech), ZFP36 (1:50, 12737-1-AP, Proteintech), Zbtb16 (1:50, F2699, Selleck), and β-actin (1:50, 66009-1-Ig, Proteintech). Quantitative analysis of the protein bands was conducted using Compass software (version 6.1.0).

### 2.8 Ultrastructural analysis of myelin via transmission electron microscopy

Fresh tissue samples were trimmed into 1 mm³ cubes and immediately fixed in TEM fixative at 4 °C. After fixation, the samples were washed three times with 0.1 M phosphate buffer (PB, pH 7.4). Post-fixation was performed with 1% osmium tetroxide in 0.1 M PB for 2 hours at room temperature, followed by another three washes with PB.

Dehydration was carried out using a graded ethanol series (30%, 50%, 70%, 80%, 95%, and 100%) and acetone, each step lasting 20 minutes. The samples were then infiltrated and embedded in EMBed 812 resin via a graded acetone–resin mixture, followed by polymerization at 65 °C for 48 hours.

Ultrathin sections (60–80 nm) were cut using an ultramicrotome and collected on 150-mesh copper grids. The sections were stained with 2% uranyl acetate for 8 minutes and 2.6% lead citrate for 8 minutes, then observed under a transmission electron microscope for imaging. Myelin sheath number was quantified by counting all clearly visible myelinated axons in three non-overlapping fields per animal at 500× magnification.

Imaging of layer V of the primary visual cortex was performed at 500× magnification for each animal. Three non-overlapping, representative fields of view were selected for analysis from the layer V region of V1 in each animal. A researcher completely blinded to experimental groupings manually counted all axonal cross-sections with clear. Prior to formal analysis, counting researchers underwent specialized training using practice image sets against established inclusion criteria. Intact myelin sheaths within each field of view using ImageJ software. The final myelin count per animal was calculated as the mean of these three field counts, subsequently employed for intergroup statistical comparisons.

### 2.9 Statistical analysis

In this study, all statistical analyses were performed using SPSS (version 26.0) and R (version 4.3.1) software, with results presented as mean ± standard deviation (SD). Graphs were generated using GraphPad Prism 9.0 software. Baseline values for the NC, FDM, and REC groups were assessed via one-way analysis of variance (ANOVA), followed by Tukey’s *post hoc* HSD test for multiple comparisons between groups to control the family-wise error rate. A p-value < 0.05 was considered statistically significant.

## 3 Results

### 3.1 Visual deprivation and recovery drive reversible changes in ocular structure and cortical function

Form deprivation successfully induced myopia in guinea pigs, as indicated by significant axial elongation and a myopic refractive shift after 4 weeks in both the FDM and REC groups compared with the NC group (**Figures 1B–D**). By the experimental endpoint (Week 5), the continuously deprived FDM group exhibited the most pronounced phenotype, characterized by the greatest axial length and the highest degree of myopia. Consistent with previous reports (30), the REC group, which underwent visual restoration after 4 weeks of deprivation, displayed a marked reversal of these changes, showing a slower rate of axial elongation and a significant reduction in myopic refraction relative to the FDM group. Functional evaluation using visual evoked potentials (VEPs) further revealed corresponding impairments in cortical responses. The FDM group showed a significant decrease in P2 amplitude and a prolonged P2 latency at Week 5 (**Figures 1E–G**), indicating delayed and weakened visual processing. Notably, these electrophysiological deficits were partially rescued in the REC group, which demonstrated significant recovery of both P2 parameters toward normal levels, suggesting functional restoration of the visual pathway following visual input recovery (31).

### 3.2 Non-neuronal cell populations in the visual cortex undergo deprivation-induced shifts

**Figure 2:**
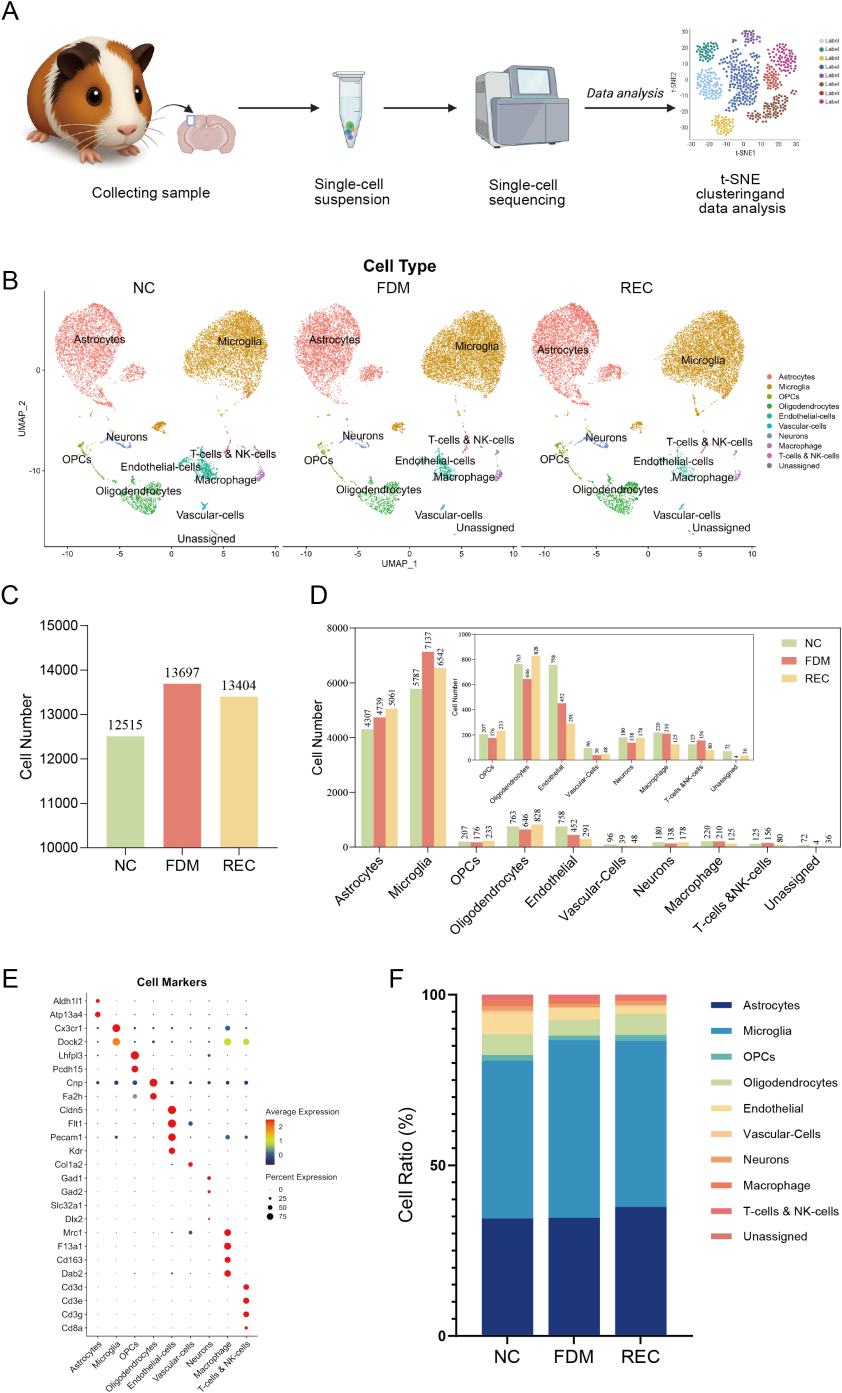
10x single-cell transcriptome analysis of non-neuronal cells in guinea pigs’ V1 across NC, FDM, REC groups. **(A)** Schematic workflow of the single-cell RNA sequencing (scRNA-seq) study. **(B)** UMAP visualization of all captured and annotated cells from the V1 of NC, FDM, and REC groups. **(C)** Total number of cells captured per group. **(D)** Bar graph showing the absolute number of cells for each annotated cell type per group. **(E)** Dot plot displaying the expression of canonical marker genes used for cell type annotation. **(F)** Bar graph showing the relative proportion (percentage) of each major cell type within the captured population per group.

To comprehensively characterize the cellular transcriptome of the primary visual cortex (V1), we performed 10x Genomics single-cell RNA sequencing (scRNA-seq). The experimental workflow, from tissue dissociation to bioinformatic analysis, is summarized in **Figure 2A**. Cell type classification was performed based on previously established V1 datasets and canonical marker genes reported in earlier scRNA-seq studies (10, 32–34).

Non-neuronal cells were robustly segregated into distinct clusters consistent with known cell identities (**Figure 2B**). Cluster annotation was confirmed using conserved, cross-species marker genes (**Figure 2E**), ensuring the validity of comparative analyses across mammalian models (35). The identified non-neuronal populations included microglia, astrocytes, oligodendrocytes, oligodendrocyte precursor cells (OPCs), endothelial cells (ECs), vascular cells (VCs), smooth muscle cells (SMCs), macrophages, and T cells. Immune-related cells, such as macrophages and T cells, were detected in low abundance, consistent with their limited representation in the visual cortex.

We next examined how visual manipulation influenced the abundance and composition of these non-neuronal cell types. The total number of cells captured from V1 is shown in **Figure 2C**. Quantitative comparison of cellular proportions suggested cell type–specific trends across groups (**Figures 2D, F**). Under form-deprivation conditions, the proportions of astrocytes and microglia appeared elevated, suggesting enhanced gliosis and immune activation. In contrast, the proportions of oligodendrocytes and OPCs were lower, indicating a potential impairment of oligodendrocyte differentiation and myelin maintenance. Following visual recovery, microglial proportion declined, while oligodendrocyte proportion increased, consistent with partial restoration of cellular homeostasis. Notably, astrocyte numbers continued to rise during recovery, suggesting a sustained or delayed reactive response even after the deprivation stimulus was removed (36).

In summary, monocular form deprivation was associated with distinct, bidirectional trends among non-neuronal cells of the visual cortex—an increase in astrocytic and microglial alongside a decrease in the oligodendrocyte lineage. Restoration of normal vision largely reversed these alterations in microglia and oligodendrocytes, highlighting their dynamic and reversible behavior in response to sensory experience. These findings identify glial cell plasticity as a key cellular correlate of both the pathological impact of FDM and its recovery, underscoring the adaptive potential of the glial network in cortical remodeling.

### 3.3 Form deprivation triggers a pro-inflammatory transcriptional program in microglia

To elucidate how visual experience shapes microglial function, we analyzed their transcriptional profiles following FDM and subsequent visual recovery (REC). Functional enrichment analyses revealed a dynamic transition in microglial state—from a pro-inflammatory phenotype under FDM to an attenuated, homeostatic profile after recovery.

**Figure 3:**
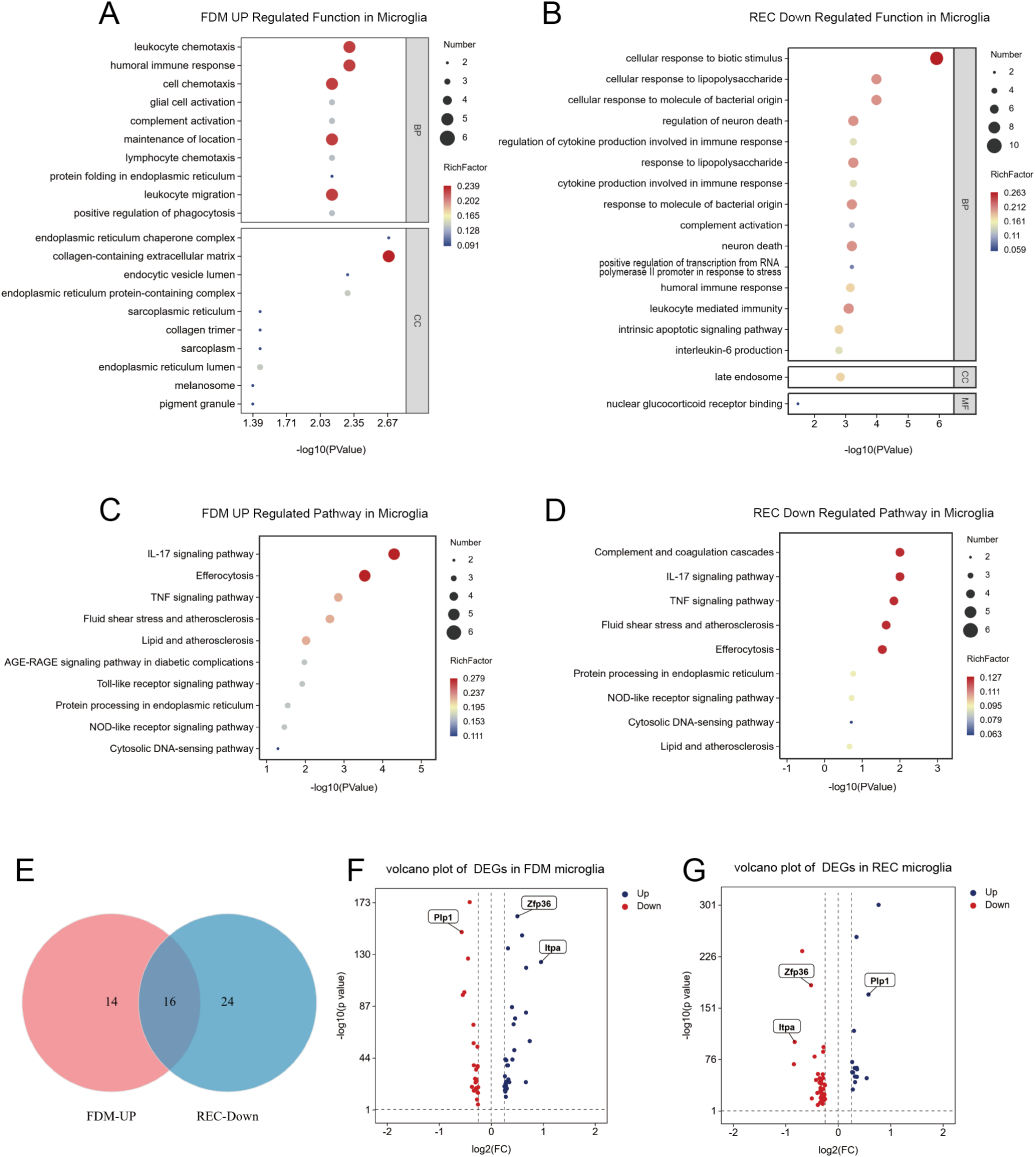
Microglia: Enrichment, Pathways & Differential Genes (FDM/REC/NC). **(A)** Top 10 enriched Gene Ontology (GO) terms for genes upregulated in FDM microglia compared to NC (adjusted P < 0.05). **(B)** Top 15 enriched GO terms for genes downregulated in REC microglia compared to FDM (adjusted P < 0.05). **(C)** Top 15 enriched KEGG pathways for genes upregulated in FDM microglia (excluding disease-related terms). **(D)** Top 15 enriched KEGG pathways for genes downregulated in REC microglia. **(E)** Venn diagram showing overlap of genes upregulated in FDM and downregulated in REC. **(F)** Volcano plot of differentially expressed genes (DEGs) in microglia: NC vs. FDM. Red dots: upregulated in FDM (log2FC > 2, adjusted P < 0.001). Blue dots: downregulated in FDM. Key genes are labeled, including ZFP36, ITPA, and the oligodendrocyte marker Plp1 (downregulated in FDM). **(G)** Volcano plot of DEGs in microglia: FDM vs. REC. Red dots: upregulated in FDM (log2FC > 2, adjusted P < 0.001). Blue dots: downregulated in FDM (i.e., upregulated in REC). Key genes are labeled, including ZFP36, ITPA, and Plp1 (upregulated in REC).

In the FDM group, microglia exhibited a robust inflammatory activation. Gene Ontology (GO) enrichment indicated significant upregulation of immune-related processes essential for inflammation initiation and propagation, including leukocyte chemotaxis, humoral immune response, and glial activation (**Figure 3A**). Consistently, KEGG pathway analysis identified strong enrichment of canonical pro-inflammatory cascades, such as the IL-17, TNF, and Toll-like receptor signaling pathways (**Figure 3C**).

Following visual restoration, this inflammatory transcriptional signature was markedly diminished. GO analysis revealed significant downregulation of immune response–associated pathways, including cellular responses to lipopolysaccharide and cytokine production (**Figure 3B**). Notably, the IL-17 signaling pathway, which was strongly activated during deprivation, was substantially suppressed after recovery (**Figure 3D**), underscoring its dynamic modulation in concert with the phenotypic reversal of microglia (37).

At the gene level, a Venn diagram analysis identified 36 genes upregulated in FDM but downregulated in REC (**Figure 3E**). Among these, *ZFP36* and *ITPA* emerged as key inversely regulated candidates (**Figures 3F, G**), suggesting potential regulatory roles in inflammation resolution. Notably, the expression of *Plp1*—a gene encoding the major myelin protein essential for axonal support (38)—was also dynamically regulated, being downregulated in FDM and upregulated in REC (**Figures 3F, G**). This pattern aligns with the observed ultrastructural myelin deficits and recovery, further linking the microglial inflammatory state to oligodendrocyte integrity.

Collectively, these findings demonstrate that form deprivation induces a pronounced pro-inflammatory state in cortical microglia, which is largely reversed upon the restoration of normal visual experience, highlighting the plastic and reversible nature of microglial responses to sensory input.

### 3.4 Activated microglia adopt a reactive morphology and molecular signature

**Figure 4:**
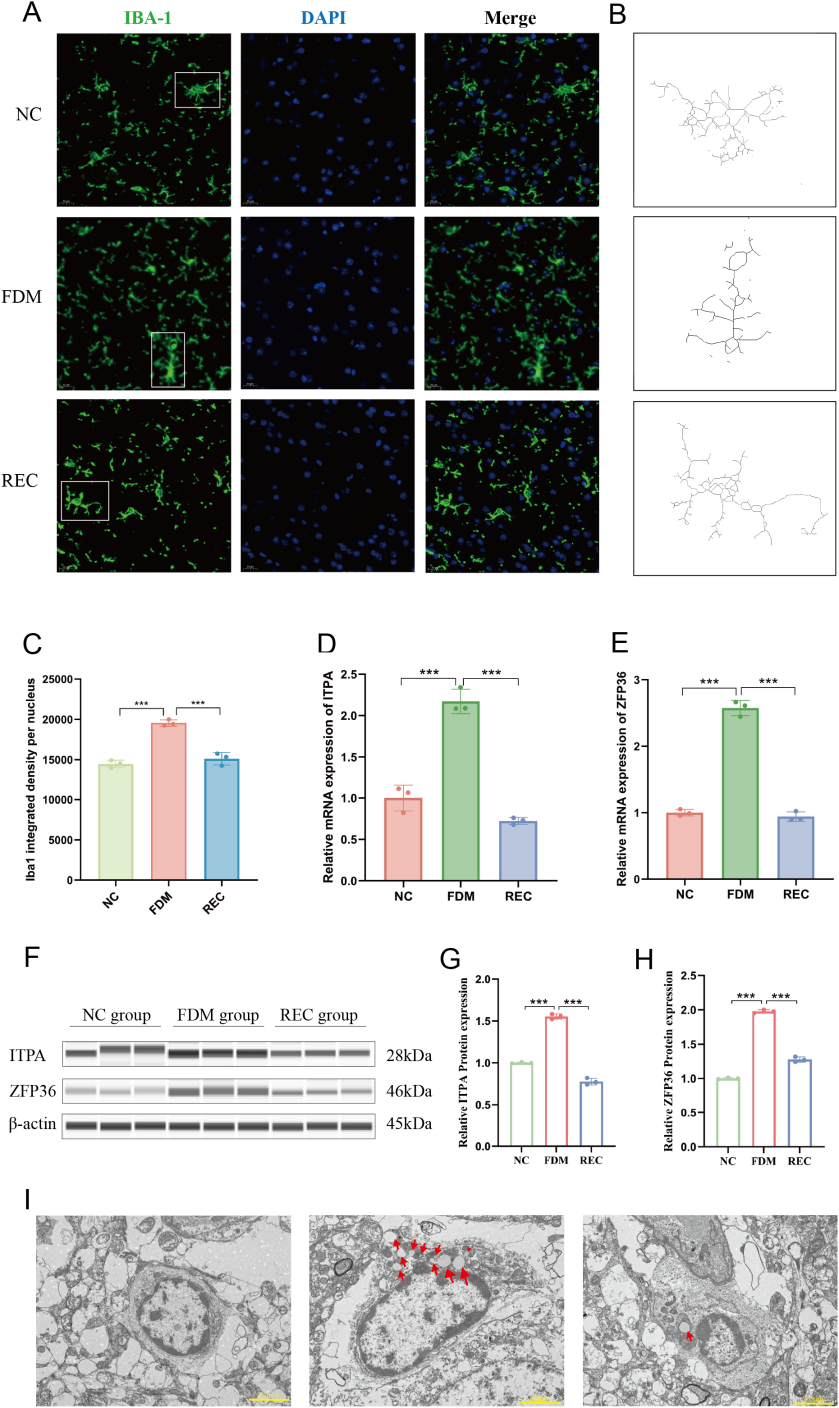
Expression of microglial markers and ITPA/ZFP36 in NC, FDM, and REC groups. **(A)** Representative immunofluorescence images of Iba1+ microglia (green) and DAPI (blue) in V1. Scale bar: 20 μm. Three non-overlapping fields per animal (n = 3 animals per group) were randomly selected for imaging; all acquisition parameters were kept constant. **(B)** Reconstructed microglial skeletons showing morphological changes: ramified in NC, activated (hypertrophied) in FDM, and restored ramification in REC. **(C)** Quantification of Iba1+ cell density. Data are mean ± SEM with individual data points (n = 3 animals per group, 3 fields per animal). ***P < 0.001, one-way ANOVA with Tukey’s HSD test. **(D, E)** qPCR validation of ITPA **(D)** and ZFP36 **(E)** mRNA expression (n = 3 per group). Data are normalized to β-actin and presented as fold change relative to NC. ***P < 0.001, one-way ANOVA with Tukey’s HSD test. **(F)** Representative capillary Western blot images for ITPA, ZFP36, and β-actin (loading control). **(G, H)** Densitometric quantification of ITPA **(G)** and ZFP36 **(H)** protein levels normalized to β-actin (n = 3 per group). Data are mean ± SEM with individual points. ***P < 0.001, one-way ANOVA with Tukey’s HSD test.

To validate the transcriptomic findings at the cellular and molecular levels, we performed immunofluorescence, morphological assessment, qPCR, Western blotting, and electron microscopy. Immunofluorescence staining for the microglial marker IBA-1 revealed a significant increase in microglial abundance in the FDM group compared with the NC group, which was markedly reduced after recovery (**Figures 4A, C**), indicating microglial proliferation during deprivation and regression upon restoration of normal vision.

Morphological reconstruction of microglial skeletons further demonstrated a pronounced phenotypic transition. In the FDM group, microglia displayed typical activated morphologies characterized by enlarged somata and retracted, thickened processes (39). In contrast, microglia in the REC group largely regained a highly ramified structure comparable to the NC group, reflecting a return to a resting state (**Figure 4B**).

We next verified the expression patterns of the key genes ITPA and ZFP36, identified from the scRNA-seq analysis. qPCR confirmed significant upregulation of both genes in the FDM group and downregulation in the REC group (**Figures 4D, E**). Western blot analysis corroborated these trends at the protein level, revealing parallel changes in ITPA and ZFP36 expression (**Figures 4F–H**).

Finally, ultrastructural examination by transmission electron microscopy (TEM) revealed abundant lipid droplets within microglia from the FDM group—a hallmark of inflammatory activation (40). Notably, these lipid inclusions were substantially reduced following visual recovery (**Figure 4I**), providing ultrastructural evidence of inflammation resolution consistent with the transcriptional and molecular findings.

### 3.5 Zbtb16 mediates experience-dependent oligodendrocyte differentiation and myelination

**Figure 5:**
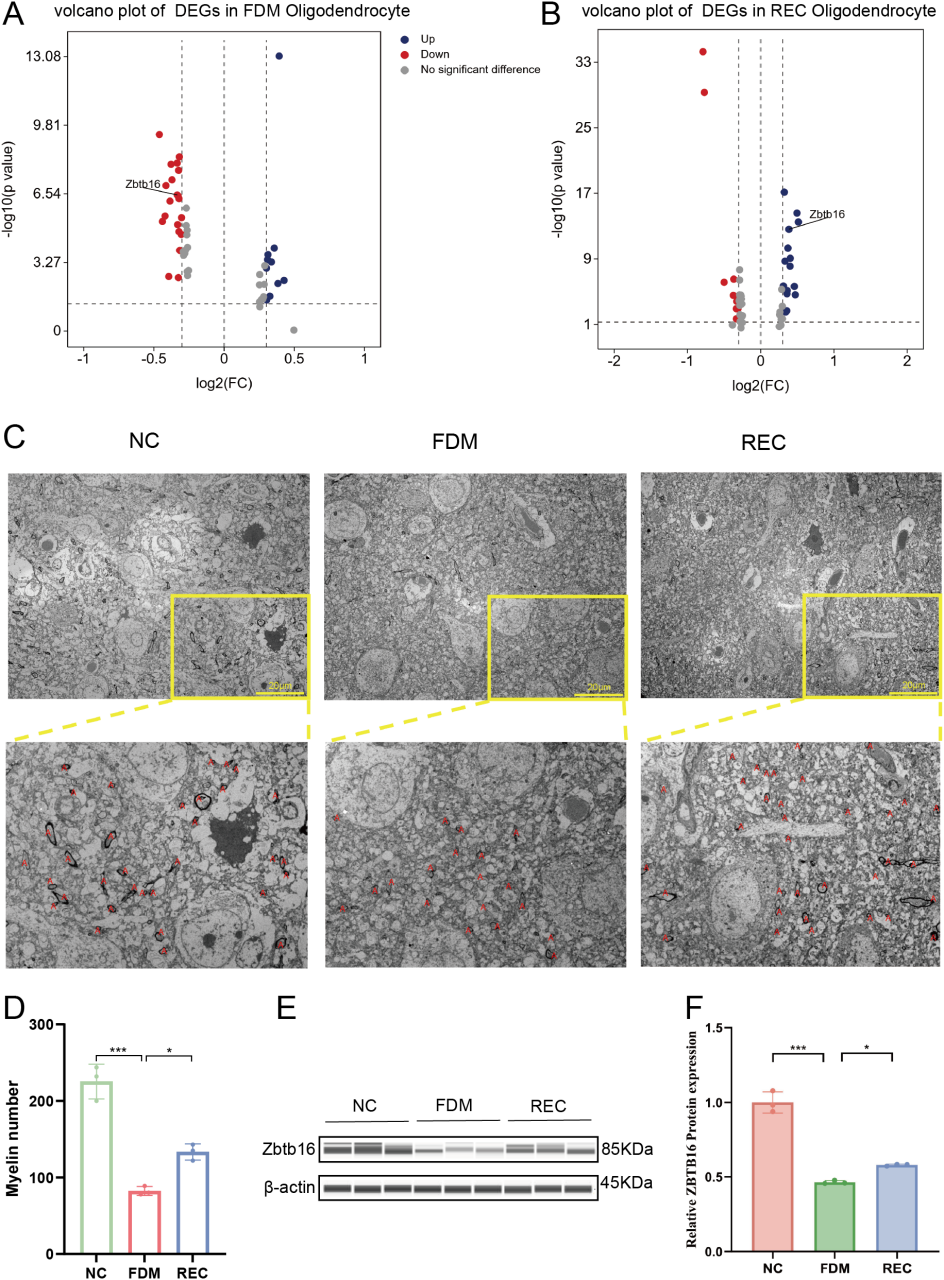
Zbtb16 mediates experience-dependent oligodendrocyte differentiation and myelination. **(A)** Volcano plot of DEGs in oligodendrocytes: NC vs. FDM. Zbtb16 is highlighted (downregulated in FDM). Criteria: |log2FC| > 2, adjusted P < 0.001. **(B)** Volcano plot of DEGs in oligodendrocytes: FDM vs. REC. Zbtb16 is highlighted (upregulated in REC). **(C)** Representative transmission electron microscopy images of myelinated axons in V1. Scale bar: 2 μm. **(D)** Quantification of myelin sheath number per field, counted according to a standardized, blinded protocol (n = 3 animals per group). Data are mean ± SEM with individual points. ***P < 0.001, *P<0.05, one-way ANOVA with Tukey’s HSD test. **(E)** Representative Western blot for Zbtb16 and β-actin. **(F)** Quantification of Zbtb16 protein expression normalized to β-actin (n = 3 per group). Data are mean ± SEM with individual points. ***P < 0.001, *P<0.05, one-way ANOVA with Tukey’s HSD test.

Oligodendrocytes (OLs) play a critical role in modulating visual pathway signaling and cortical plasticity through myelination; however, the molecular mechanisms underlying their regulation remain incompletely understood. Previous studies have shown that *Zbtb16* knockout mice exhibit impaired OL differentiation and hypomyelination (41). Consistent with these findings, our single-cell transcriptomic analysis revealed a marked downregulation of *Zbtb16* expression in OLs from the FDM group (**Figure 5A**), accompanied by a significant reduction in OL abundance. Importantly, *Zbtb16* expression was restored in the REC group (**Figure 5B**), suggesting that its suppression may contribute to OL dysfunction during form deprivation and its reactivation may facilitate remyelination during recovery.

To determine whether *Zbtb16* downregulation was associated with structural myelin deficits, we performed transmission electron microscopy (TEM) of the primary visual cortex (42). In the FDM group, myelin sheaths appeared sparse and thinner compared with the NC group, indicative of hypomyelination. By contrast, the REC group exhibited clear ultrastructural restoration, with compact and continuous myelin lamellae (**Figure 5C**). Quantitative analysis confirmed a significant reduction in myelin number and thickness in the FDM group, both of which were substantially recovered after visual restoration (**Figure 5D**). We next sought to confirm the observed transcriptional changes at the protein level. Western blot analysis revealed that *Zbtb16* protein expression was significantly suppressed in the FDM group, mirroring the scRNA-seq results. Following visual recovery, *Zbtb16* expression showed a significant increase, a trend that paralleled the restoration of myelin integrity and cortical function (**Figures 5E, F**). This concordance at the protein level solidifies *Zbtb16* as a key experience-dependent regulator of oligodendrocyte differentiation.

Collectively, these results suggest that *Zbtb16* downregulation contributes to OL loss and impaired differentiation, leading to myelination deficits in the visual cortex during form deprivation. Restoration of *Zbtb16* expression upon normal visual experience likely underlies the structural and functional recovery of cortical myelin.

### 3.6 Pseudotime and cell communication analyses reveal a reversible blockade in oligodendrocyte differentiation

**Figure 6:**
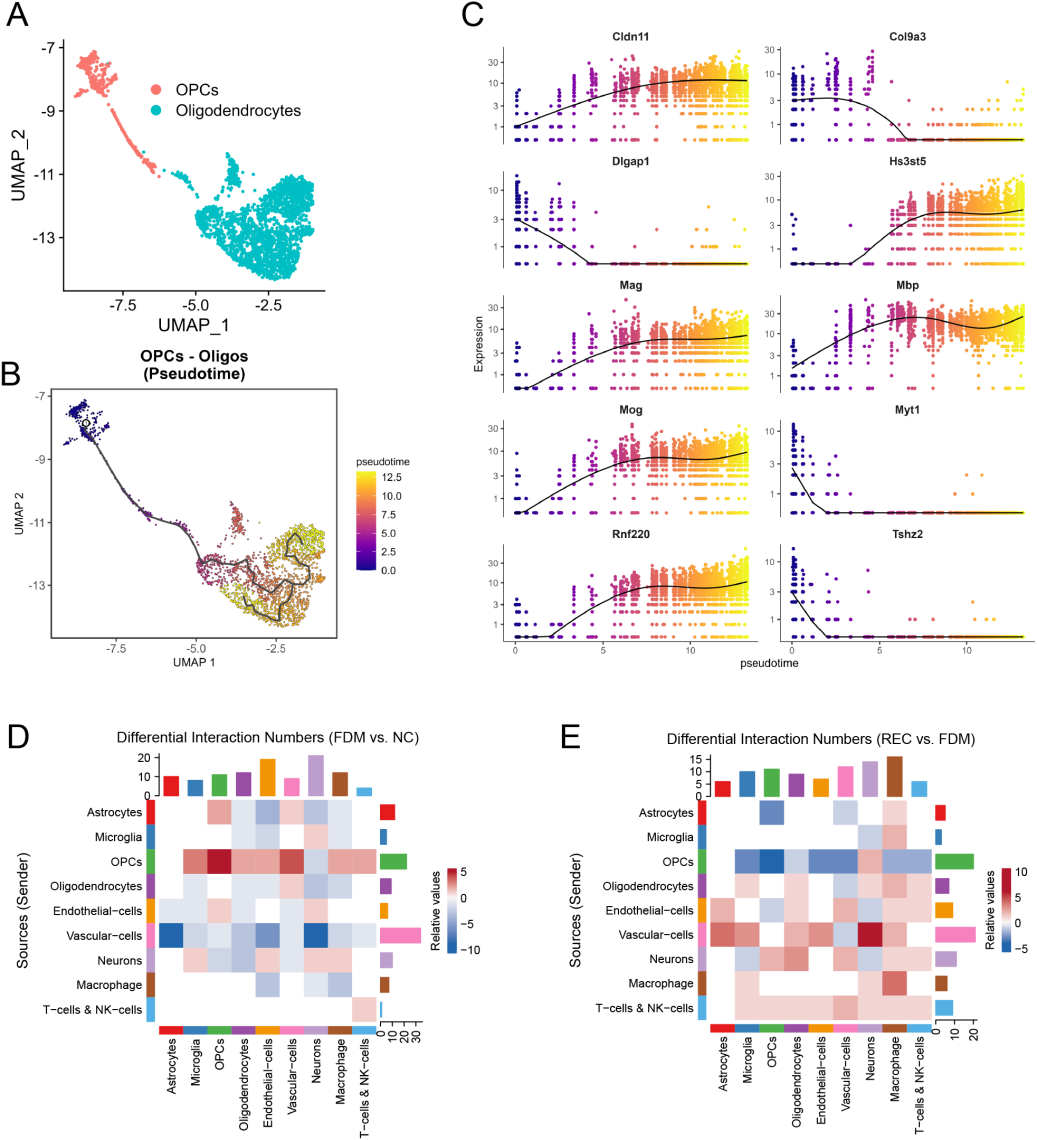
Pseudotime Analysis of Oligodendrocytes and Differential Cell Communication in FDM/REC Groups. **(A)** UMAP showing oligodendrocyte lineage clusters: OPCs and mature oligodendrocytes (OLs). **(B)** Pseudotime trajectory inferred by Monocle3, showing progression from OPCs to OLs. **(C)** Dynamic expression of key myelin-related genes (Mbp, Mag, Mog) and differentiation regulator Myt1 along pseudotime. **(D)** Heatmap showing differential numbers of cell–cell interactions between FDM and NC groups. Red: increased interactions in FDM; blue: decreased. **(E)** Heatmap showing differential numbers of cell–cell interactions between REC and FDM groups, highlighting restoration of normal communication patterns.

Pseudotime analysis of the oligodendrocyte lineage revealed a clear differentiation trajectory from OPCs to mature OLs (**Figures 6A, B**). Key myelin-related genes (e.g., Mbp, Mag, Mog) were upregulated late in pseudotime, while differentiation regulators like Myt1 were downregulated (**Figure 6C**), indicating a transcriptional blockade in OL maturation under FDM. Cell communication analysis provided insights into the altered microenvironment contributing to this blockade. Comparative analysis between FDM and NC groups revealed a broad increase in predicted signaling interactions. Of particular note, we observed a pronounced enhancement in signaling from microglia to OPCs (**Figure 6D**), coinciding with the period of differentiation arrest.

This perturbation in cellular crosstalk, especially the heightened microglia-to-OPC communication, was substantially reversed upon visual recovery. The REC group showed a global reduction in these elevated interactions, with signaling from microglia to OPCs being significantly diminished compared to the FDM group (**Figure 6E**). The restoration of a more homeostatic communication pattern, particularly along the microglia-OPC axis, coincided with the resolution of the OL differentiation blockade and the structural repair of myelin observed in our pseudotime and ultrastructural analyses.

## 4 Discussion

**Figure 7:**
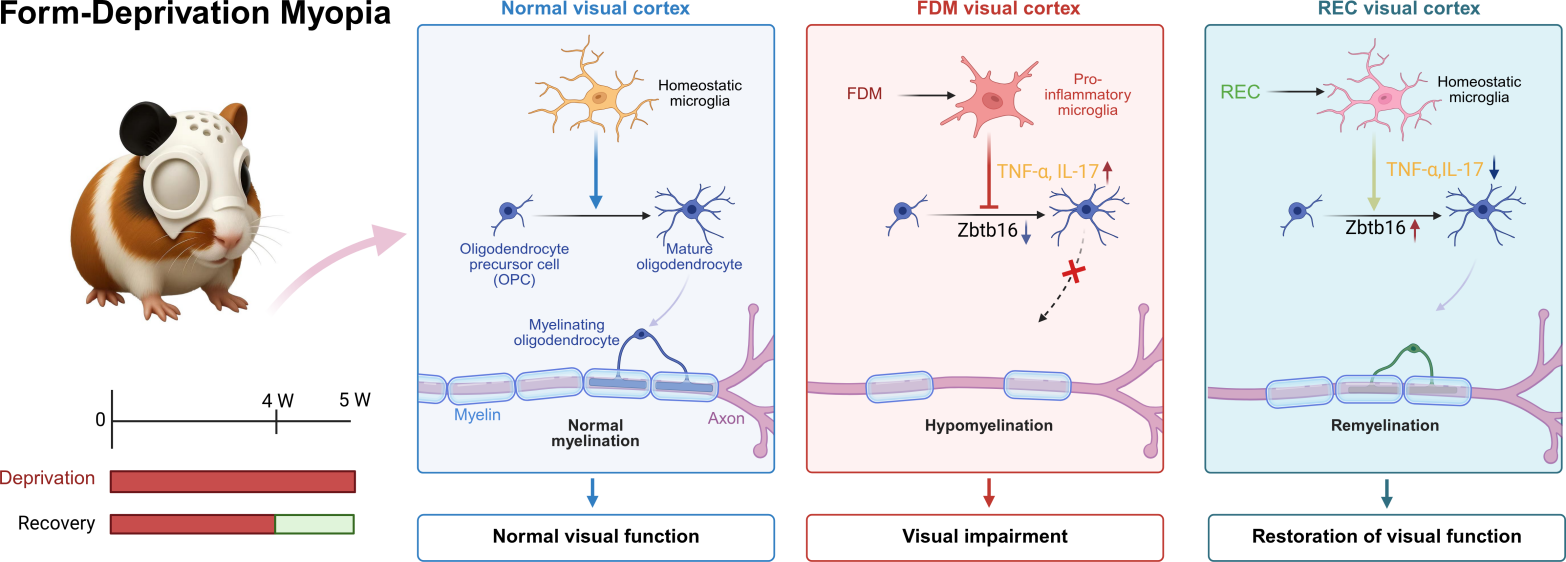
Reversible changes in the visual cortical microglia–oligodendrocyte axis following deprivation and recovery. This diagram summarizes the reversible remodeling of the microglia–oligodendrocyte axis in the visual cortex during deprivation and recovery. Normal (Left): Microglia in homeostasis support oligodendrocyte differentiation and myelination, enabling normal vision. FDM (Middle): Monocular deprivation activates microglia (↑TNF-α/IL-1β) and downregulates Zbtb16, impairing oligodendrocyte differentiation and myelination, leading to visual deficits. REC (Right): Visual restoration reverses microglial activation, restores Zbtb16 expression, and promotes remyelination and functional recovery.

Research on FDM has traditionally centered on ocular mechanisms. However, how aberrant visual input influences cortical cellular circuits and ultimately impairs visual processing remains a critical yet underexplored question. By integrating single-cell transcriptomics with structural and functional analyses, our study uncovers a pivotal role of glial cells—particularly microglia and OLs—within the primary visual cortex (V1) in mediating this process. Our data suggest that visual deprivation is associated with a reversible neuroimmune cascade involving microglia: microglia adopt a pro-inflammatory phenotype and show increased expression of cytokines such as TNF-α, which coincides with downregulation of key OL differentiation regulators, including *Zbtb16*, leading to hypomyelination and altered visual evoked potentials. The identification of this “microglia–oligodendrocyte axis” broadens the concept of cortical plasticity beyond neuronal circuits to encompass the dynamic and reciprocal interactions among glial populations (**Figure 7**).

Our findings extend the established role of microglia from immune surveillance to active regulation of experience-dependent cortical plasticity (43). A central discovery is the identification of a highly reversible glial axis operating during visual deprivation and recovery. Visual deprivation is accompanied by a pronounced pro-inflammatory transformation in microglia, which is rapidly reversed upon restoration of normal visual input as their transcriptional state returns to homeostasis. This recovery, consistent with observations by Kaneko et al. (14), is mechanistically linked to glial transcriptional reprogramming. Following visual restoration, key inflammatory pathways (e.g., IL-17, TNF-α) were markedly downregulated, and both microglial and oligodendroglial transcriptomes reverted toward homeostatic profiles, demonstrating the remarkable adaptability of the cortical glial network. Thus, the expression level of myelin structural proteins, such as *Plp1*, serves as a sensitive indicator of oligodendrocyte functional status and cortical circuit maturity. Its dynamic regulation provides critical insight into the mechanisms underlying experience-dependent myelin remodeling. We further identify Zbtb16 as an experience-dependent transcriptional hub that is associated with microglia–oligodendrocyte interactions (41). Collectively, these results define a dynamic, reversible glial axis linking neuroimmune signaling to white matter plasticity and cortical circuit remodeling in response to altered sensory input.

Oligodendrocyte differentiation and myelination capacity were likewise dynamically and reversibly altered in parallel with changes in upstream microglial signaling (44). Molecular evidence indicates that hypomyelination coincides with a differentiation blockade. Pseudotime analysis revealed that OPCs failed to progress to late developmental stages characterized by high expression of myelin genes (Mbp, Mag, Mog) (45), explaining the observed ultrastructural deficits. Visual recovery relieved this blockade, enabling normal progression, gene upregulation, and restoration of myelin integrity—demonstrating robust plasticity within the oligodendrocyte lineage.

Aberrant visual experience is associated with microglial activation and pro-inflammatory signaling that coincides with suppressed oligodendrocyte differentiation; conversely, recovery is associated with resolution of inflammation and remyelination. Cell communication analyses further indicate that visual restoration remodels the broader microglia–oligodendrocyte signaling network, transforming the cortical microenvironment from inhibitory to reparative. This finding extends the framework proposed by Miron (24), highlighting the reversibility and bidirectional nature of microglial involvement in the oligodendrocyte lineage dynamics. Together, these results reveal an intrinsic homeostatic mechanism within the visual cortical glial network that is associated with restoration of cellular and functional balance following sensory perturbation.

Despite these insights, several limitations of this study should be acknowledged. First, while we demonstrate a strong co-variation between microglial activation and OL differentiation status, causality remains to be directly established. Functional confirmation will require *in vivo* manipulation of microglia (20)—such as selective depletion or inhibition of inflammatory cytokines (e.g., TNF-α)—to test whether such interventions can rescue OL differentiation and myelination. Second, although single-cell RNA sequencing provides a high-resolution transcriptional landscape, it lacks spatial resolution. Thus, the predicted microglia–OL interactions are currently inferred from ligand–receptor co-expression rather than direct spatial proximity. Future spatial transcriptomics or *in situ* imaging studies will be essential to confirm physical and functional coupling between activated microglia and differentiation-blocked OPCs. Third, while key molecules such as *ZFP36 (*46), *ITPA (*47), *Zbtb16 (*41) and *Plp1* exhibit expression dynamics that tightly correlate with the FDM phenotype, these associations remain correlative. Due to technical limitations in our model, we were unable to perform cell type–specific genetic manipulation (e.g., conditional knockdown or overexpression) to directly test their causal roles. Hence, these factors represent high-priority candidates for future mechanistic dissection. It should be noted that this study was conducted in young guinea pigs (2–7 weeks old), a period of high neuronal and glial plasticity. The robust recovery observed may therefore reflect the enhanced plasticity of the developing visual system. Future studies in adult or aged animals will be necessary to determine whether similar reversible glial reprogramming occurs beyond the critical period.

The central implication of our study is that the pathophysiology of myopia extends beyond the eye to the cortical level, where glial networks serve as key mediators of experience-dependent plasticity. Our results show an association between microglial inflammatory states and OL differentiation: aberrant visual input is accompanied by pro-inflammatory signaling (notably via TNF-α), which coincides with suppresses *Zbtb16* expression in OLs and impaired myelination. Restoration of normal vision is associated with reversal of this process, reflecting an intrinsic homeostatic capacity within the cortical glial network. This finding reframes the visual cortex as an active participant in the adaptive and maladaptive responses to altered sensory experience, rather than a passive downstream recipient of retinal input.

Based on our results, we propose a mechanistic model wherein during critical developmental periods, the quality of visual experience regulates cortical myelination by modulating microglial inflammatory tone. Microglia thus act as pivotal transducers, translating environmental stimuli into molecular programs that are associated with OL differentiation and circuit maturation. In this framework, the microglia–OL axis constitutes a central mechanism linking neuroimmune activity to experience-dependent plasticity and cortical circuit refinement.

Looking ahead, future work should focus on experimentally dissecting the molecular pathways operating within this glial axis. Targeting regulators such as *ZFP36* and *ITPA* in microglia could modulate their inflammatory profile and thereby enhance OL differentiation and remyelination. Similarly, manipulating *Zbtb16* expression in OLs may offer a route to restore normal myelination in pathological conditions associated with aberrant visual experience. Such approaches hold translational potential for developing therapeutic interventions aimed at mitigating central nervous system alterations in myopia and possibly other sensory-deprivation–related disorders.

## 5 Conclusions

In summary, our study demonstrates that the primary visual cortex undergoes extensive glial reprogramming in response to altered sensory experience. We identify a pivotal microglia–oligodendrocyte axis that is associated with visual deprivation, microglial activation and pro-inflammatory signaling, which in turn coincides with disrupted oligodendrocyte differentiation and hypomyelination. Importantly, this pathological cascade is fully reversible: restoration of normal visual input is associated with attenuation of microglial inflammation, reinstatement of oligodendrocyte differentiation and myelin gene expression, and both structural and functional recovery.

These findings broaden the pathophysiological framework of myopia beyond ocular mechanisms to include maladaptive plasticity within central visual circuits. They establish glial cells—notably microglia and oligodendrocytes—as active, experience-dependent regulators of cortical function rather than passive support elements. The reversibility of this microglia–oligodendrocyte axis highlights its therapeutic potential as a target for interventions aimed at enhancing visual rehabilitation and restoring cortical homeostasis following sensory deprivation.

## Data Availability

The data presented in this study have been deposited in the NCBI repository under accession number PRJNA1421994.
